# The largest fish in the world’s biggest river: Genetic connectivity and conservation of *Arapaima gigas* in the Amazon and Araguaia-Tocantins drainages

**DOI:** 10.1371/journal.pone.0220882

**Published:** 2019-08-16

**Authors:** Izeni Pires Farias, Stuart Willis, Adam Leão, Júlia Tovar Verba, Marcelo Crossa, Fausto Foresti, Fabio Porto-Foresti, Iracilda Sampaio, Tomas Hrbek

**Affiliations:** 1 Laboratório de Evolução e Genética Animal/LEGAL, Universidade Federal do Amazonas (UFAM), Manaus, Amazonas, Brazil; 2 Department of Ichthyology, California Academy of Sciences, San Francisco, CA, United States of America; 3 Departamento de Ecologia, Universidade Federal do Rio Grande do Norte (UFRN), Natal, Brazil; 4 Acqua Consultoria Ambiental, Rio de Janeiro, Brazil; 5 Laboratório de Biologia e Genética de Peixes, Instituto de Biociências, Universidade Estadual Paulista (UNESP), Botucatu, São Paulo, Brazil; 6 Departamento de Ciências Biológicas, Faculdade de Ciências, Universidade Estadual Paulista (UNESP), Campus de Bauru, Bauru, Brazil; 7 Instituto de Estudos Costeiros, Universidade Federal do Pará, Campus Universitário de Bragança, Pará, Brazil; DePaul University, UNITED STATES

## Abstract

Arapaima, pirarucu or paiche (*Arapaima gigas*) is one of the largest freshwater fish in the world, and has a long history of commercial exploitation in the Amazon region. To estimate levels of genetic variability and historical and recent connectivity in Arapaima, we examined variation in eleven microsatellite DNA markers in individuals from 22 localities in Brazil, Colombia, and Peru. The results of analysis of molecular variance, Bayesian clustering and discriminant analysis of principal components showed that *Arapaima* in our samples represents two major populations, one in the Amazonas and one in the Araguaia-Tocantins River basins. The Amazonas population is further structured by isolation-by-distance with the hydrologically largely unconnected Amapá locality representing the eastern-most extreme of this continuum; gene flow predominates at distances of less than 1500 km with localities separated by over 2000 km dominated by genetic drift and effectively forming different populations. We saw no evidence of multiple species of *Arapaima* in the Amazonas basin, and analysis of pairwise genetic divergence (*F*_*ST*_) with Mantel tests and correlograms indicated that this largest population exhibits a large-scale pattern of isolation-by-distance, with which results from MIGRATE-N agreed. The degree and significance of genetic divergence indicates that most sampled localities represent demographically independent sub-populations, although we did identify several recent migration events between both proximal and more distant localities. The levels of genetic diversity were heterogeneous across sites, including low genetic diversity, effective population sizes, and evidence of genetic bottlenecks in several places. On average the levels of gene diversity and rarefied allelic richness were higher for localities along the Amazonas mainstem than in the tributaries, despite these being the areas of highest fishing pressure, while the lowest values were found in tributary headwaters, where landscape modification is a significant threat. We recommend that managers consider the regional and local threats to these populations and tailor strategies accordingly, strategies which should ensure the ability of young *A*. *gigas* to disperse through floodplain corridors to maintain genetic diversity among otherwise sedentary adult sub-populations.

## Introduction

The Amazon basin suffers from the myth of superabundance, wherein the natural resources present in this region, including its impressive diversity of fishes, are considered inexhaustible by any human demand [1, 2]. This myth derives in part from the huge scale of the Amazon, its high biodiversity, and its low relative human occupational density, since the perceived abundance of a region’s natural resources is directly related to the intensity of exploitation [2]. Indeed this myth of superabundance has been promulgated since the Portuguese colonized the Amazon basin, and particularly during the “boom” of latex rubber export [3]. However, there are well documented cases of drastic reductions in exploited populations, including the black (*Melanosuchus niger*) and spectacled caimans (*Caiman crocodilus*) [4, 5], the Amazonian manatee (*Trichechus inunguis*) [6], numerous species of turtles (*Podocnemis* spp.) [7], and what may be South America’s largest freshwater fish, the arapaima, pirarucu or paiche (*Arapaima gigas* Schinz 1822) [8]. Although the iconic arapaima continues to be exploited, it is described on the IUCN Red List as Data Deficient (IUCN, 2017), meaning there is insufficient knowledge of its biology, ecology, and genetics to effectively manage its conservation.

Another prominent uncertainty surrounding the arapaima is the number of species present in this genus and their distribution. In two recent publications, Stewart [9, 10] revised the taxonomy of the genus, revalidated several species and described a new species of *Arapaima* from the central Amazon [9]. Stewart [9, 10] puts forth an argument that at least six species of *Arapaima* exist: 1) *Arapaima gigas* (Schinz, in Cuvier 1822) described from near [Vila] Santarém, Para State, Brazil, only known from the holotype (MNHN A.8837); 2) *Arapaima mapae* (Valenciennes, in Cuvier and Valenciennes, 1847) described from Lago do Amapá or Lago Grande in Região dos Lagos in Amapá State, Brazil, only known from the holotype (MNHN A.8836); 3) *Arapaima arapaima* (Valenciennes, in Cuvier and Valenciennes, 1847) described from Guyana (Essequibo basin), only known from the holotype (BMNH 2009.1.19.1) but the holotype is misplaced or lost; 4) *Arapaima agassizii* (Valenciennes, in Cuvier and Valenciennes, 1847) described from “Brazilian Amazon”, only known from an illustration of the holotype by Spix and Agassiz [11] and the holotype is lost; 5) *Arapaima leposoma* Steward 2013 described from one specimen collected from the Solimões River shortly upstream of the mouth of the Purus River, Amazonas State, Brazil, only known from the holotype (INPA 16847); and 6) *Arapaima* sp. *incertae sedis*, i.e. a species of uncertain taxonomic status, that apparently comprises all other Amazon basin *Arapaima* specimens deposited in scientific collections.

Günther in 1868 [12] synonymized all the species of arapaima described by Valenciennes, and Spix and Agassiz with *Arapaima gigas* (Schinz, in Cuvier 1822). In contrast to Günther [12],Stewart [9, 10] concluded that all former species should be considered valid, although they are known only from type specimens, their distributions are unknown, and all *Arapaima* specimens deposited in scientific collections—specimens sampled throughout the Amazon basin—are *Arapaima* sp. *incertae sedis*. Thus, although the specimens analyzed in this study may be *Arapaima* sp. *incertae sedis*, until further taxonomic clarification we take the conservative approach and refer to the specimens analyzed in this study as *Arapaima gigas*.

*Arapaima gigas* is one of the largest freshwater fishes in South America, competing only with the catfish *Brachplatystoma filamentosum* for that title, and can reach a length of three meters and weigh more than 200 kg [13, 14]. It is primarily piscivorous [15, 16], and the feeding habits of these large-bodied fishes provide top-down trophic regulation in floodplain ecosystems [17]. Although as adults *A*. *gigas* are primarily sedentary fish with low dispersal capability [18], they do make seasonal migrations between permanent wetlands and nearby floodplains (várzea or igapó). During the dry season, *A*. *gigas* inhabits permanent wetlands, such as slow-moving rivers and lagoons where adults develop their gonads, engage in courtship, build nests, and reproduce. During the rainy season when water levels rise, *A*. *gigas* migrate to the floodplain, where the males provide parental care including mouth brooding, and young exploit the abundant resources of the flooded zones. As the water levels fall, parental care ceases and adult *A*. *gigas* migrate back to the permanent wetlands. It is in the permanent wetlands where most fishing occurs, and *A*. *gigas* are particularly vulnerable to harpooning as these obligate air breathers surface to refresh their air bladder [19]. Individuals generally become reproductively mature after 3–5 years of age [20] and may live upwards of 15–20 years. Batch fecundity is low, however [21].

*Arapaima gigas* is native to, and historically common in, the lowland Amazonas basin and the Araguaia-Tocantins basin, the later of which is considered a separate drainage by some authors, although it is connected to the Amazonas by uninterrupted freshwater and exhibits a related biota [22]. In the late 1970’s the species was also unintentionally introduced into the Bolivian Amazon [23]. In a pioneering study of *A*. *gigas* population genetics, Hrbek et al. [24] used mitochondrial DNA sequences from 120 individuals from six sites along the main channel of the Amazon basin and one site in the Araguaia-Tocantins basin and observed greater genetic diversity (haplotype diversity) in *A*. *gigas* far from large urban centers, where arapaima meat sales and distribution centers are concentrated. These data also suggested that the effective population size of this species had declined along with known decreases in census population density after two centuries of commercial exploitation, and that the Bolivian population was introduced from the Peruvian Amazon. Later, nuclear microsatellite data from the same sites revealed a pattern of isolation-by-distance along the Amazon River main stem [25]. Similar findings were reported by Araripe et al. [26].

In the present study, we expand on previous sampling to include sites throughout the Amazon basin, including the main axis of the Amazon basin and its main tributaries, and in the Araguaia-Tocantins watershed, to examine the distribution of genetic diversity and the pattern and magnitude of population structure. We sought to test if (i) the pattern of isolation-by-distance of *A*. *gigas* populations remains throughout the Amazon and Tocantins, (ii) if population genetic structure indicated that some areas exhibited unique genetic variation indicative of historical or sustained divergence, and (iii) whether indications of declines in population genetic diversity were only present near urban centers or were evident throughout the range of this fish.

## Material and methods

### Ethics statement

Permission to collect samples of was granted by IBAMA Permit Number 11325–1.

### Sampling

**Fig 1:**
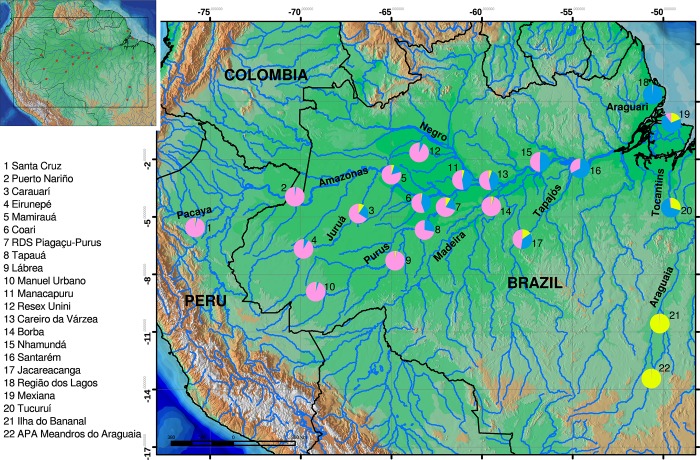
Map of collecting localities of *Arapaima gigas*. Pie plots indicate average population ancestry of each of the three main biological clusters detected in STRUCTURE analysis (see Fig 2). Color scheme is same as in Fig 2, K = 3.

We analyzed 517 individuals of *Arapaima gigas* sampled from 19 locations in the Amazon basin and three locations in the Araguaia-Tocantins basin (Fig 1 and Table 1). Samples were from natural populations and were collected by the fishing communities at each location. Tissue samples were preserved in 95% alcohol and deposited in the Coleção de Tecidos da Genética Animal (CTGA) of the Laboratório de Evolução e Genética Animal (LEGAL) at the Universidade Federal do Amazonas (UFAM) in Manaus, Brazil.

**Table 1:** Sampling areas of *Arapaima gigas* analyzed in the present study.

**Map location**	**N**	**Locality**	**River location**	**Amazon Basin**	**Coordinate Lat/Lon**
1	16	Santa Cruz	Pacaya River	Main channel	-5.50737°/-75.89130°
2	22	Puerto Nariño	Amazonas River	Main channel	-3.76613°/-70.37904°
3	18	Carauari	Middle Juruá River	Tributary	-4.93289°/-66.69848°
4	13	Eirunepé	Upper Juruá River	Tributary	-6.78775°/-69.81648°
5	32	Mamirauá	Confluence Solimões/Japurá Rivers	Main channel	-3.06471°/-64.80223°
6	9	Coari	Middle Solimões River	Main channel	-4.39622°/-63.47529°
7	20	RDS Piagaçu-Purus	Lower Purus River	Tributary	-4.14691°/-62.00704°
8	20	Tapauá	Middle Purus River	Tributary	-5.70652°/-63.20083°
9	15	Lábrea	Middle Purus River	Tributary	-7.30723°/-64.83540°
10	18	Manuel Urbano	Upper Purus River	Tributary	-8.93535°/-69.17622°
11	30	Manacapuru	Amazonas River	Main channel	-3.17434°/-60.79713°
12	21	Resex Unini	Middle Negro River	Tributary	-1.59844°/-63.41299°
13	21	Careiro da Várzea	Amazonas River	Main channel	-3.23870°/-59.89439°
14	30	Borba	Lower Madeira River	Tributary	-4.37251°/-59.52051°
15	7	Nhamundá	Lower Nhamundá River	Tributary	-2.14754°/-56.75132°
16	31	Santarém	Amazonas River	Main channel	-2.69827°/-54.90179°
17	15	Jacareacanga	Tapajós River	Tributary	-6.21984°/-57.97813°
18	30	Região dos Lagos	Araguari River	Delta periphery	1.39913°/-49.61515°
19	17	Mexiana Island	Mouth of the Amazonas River	Delta	0.01254°/-49.75654°
**Map location**	**N**	**Locality**	**River location**	**Araguaia-Tocantins Basin**	**Coordinate Lat/Lon**
20	31	Tucuruí	Tocantins River	Main channel	-4.95746°/-49.65950°
21	15	Ilha do Bananal	Araguaia River	Main channel	-10.62590°/-50.43996°
22	80	APA Meandros do Araguaia	Araguaia River	Main channel	-13.33616°/-50.77259°
Total	511				

DNA samples were extracted using the Qiagen extraction kit. Eleven microsatellite loci were amplified following Farias et al. [27] for *Arapaima gigas*. Loci used in this study were: CTm3, CTm4, CTm5, CTm7, CTm8, CAm2, CAm13, CAm15, CAm16, CAm20 and Cam26. PCR products were generated with labeled primers and visualized on a Megabace 1000 DNA automatic sequencer (GE-Healthcare). Allele sizes were scored against an internal ET-400 ROX size standard. Individuals were genotyped using the Genetic Profiler and Fragment Profiler (GE-Healthcare). MICRO-CHECKER v2.2.3 [28] was used to detect possible errors due to genotyping, null alleles or stutters. The matrix of genotypes is available at https://github.com/legalLab/publications.

### Data analysis

Various genetic diversity parameters, including the observed heterozygosity, gene diversity (expected heterozygosity) and the number of alleles per locus was estimated using Arlequin 3.5 [29]. As richness estimates are constrained by sample size [30], we implemented the rarefaction analysis in the program HP-Rare [31] so that the number of alleles and allelic richness estimates could be compared between samples localities. Heterozygosity estimates are less influenced by sample size [32], so no correction was applied. Additionally, we estimated endogamy/inbreeding coefficient (*F*_*IS*_) within each sampling site using Arlequin 3.5 [29].

To identify major population structuring patterns in the data, we utilized Bayesian clustering of individuals in the program STRUCTURE 2.3.3 [33]. This analysis clusters individual into a pre-defined number of populations (K) that minimize deviations from Hardy-Weinberg predictions and linkage disequilibrium among loci. We performed 10 independent runs for each predetermined number of biological groups (K = 1 to 22; considering that each location could be a different biological group), each run consisting of 1,000,000 MCMC chains after having discarded the first 100,000 chains as burn-in. We used the ‘admixture’ and ‘correlated-allelic-frequencies’ models with and without location information as a prior [34]. The location prior suggests that individuals sampled in the same locality are likely to belong to the same cluster, but it is considered a weak prior, while the admixture model allows individuals to have ancestry from multiple clusters. The optimal number of clusters was inferred based on changes in the precision of clustering with different K (delta K) [35]. While STRUCTURE clusters individuals, SAMOVA 2.0 [36] clusters *a priori* sampling groups (localities) into a pre-defined number of groups (K) to maximize the genetic variance among groups in a hierarchical AMOVA framework [37]. We clustered localities using 10,000 permutations from 100 starting groupings for K = 1 to 10 (upper value of K guided by STRUCTURE results) both with and without explicit geographical information. We quantified the genetic variance and significance of the STRUCTURE and SAMOVA clusterings using hierarchical AMOVA in Arlequin 3.5 [29] using 10,000 permutations with genetic distance based on allele identity. Finally, we also used a multivariate ordination approach implemented in the Discriminant Analysis of Principal Components (DAPC) [38] using the R package Adegenet 2.1 [39] in R [40]. This procedure uses a discriminant analysis (DA) to maximize the among-group variance in components from a principal components analysis (PCA) of samples assigned to pre-defined groupings (here, sampling localities). This DAPC retained 22 PC axes and 4 discriminant axes.

### Historical and contemporaneous gene flow and demography

We looked for additional spatial patterns of gene flow by testing for isolation-by-distance through correlation of genetic and geographic distances using the Mantel test [41] implemented in Arlequin 3.5 [29]. Here, geographical distances, in km, followed the course of rivers, and the genetic distance matrix contained Slatkin linear pair-wise *F*_*ST*_ values based on allele identity. The geographical distance between the sampling localities was estimated by tools available on Google Earth, version 4.2 beta 2008 (Google). We also calculated a Mantel correlogram using the *vegan* package 2.4–5 [42] in R. Distance classes used in the Mantel correlogram ranged from 0 to 5500 km in steps of 500 km, and 5500+ km. This analysis allows for discrimination of migration-dominated and genetic drift-dominated evolutionary processes across spatial scales.

Additionally, we estimated historical and recent gene flow. First, gene flow was estimated by Bayesian analysis using MIGRATE-N version 3.6.11 [43]. Recent dispersal or migration was estimated using, STRUCTURE, and by population assignment in GENODIVE 2b27 [44]. For MIGRATE-N we ran 10 short chains, sampling each chain 10,000 times. We then sampled 500,000 topologies from one long chain, discarding the first 5,000 samples as burn-in. Search of parameter space was improved though adaptive swapping among four heated chains. MIGRATE analyses were repeated three times with random seeds to assess convergence.

We used population assignment in STRUCTURE and GENODIVE v2b27 [44] to identify recent migrants. In STRUCTURE, we specified the population (sample site) origin of each individual, and estimated the probably of assignment to that cluster back two generations (USEPOPINFO = 1, GENSBACK = 2), with three different migration priors: 0.1, 0.05, and 0.01. Convergence of this chain was rapid, so we ran the analysis for 100k generations after equal burn-in, and made three replicate runs. Migrants were identified as individuals with probability less than 0.5 of being from their sampled site. In GENODIVE, we specified an alpha of 0.002 (0.05/22 populations) applied independently to each population, with those exceeding the likelihood ratio threshold identified as migrants. STRUCTURE and GENODIVE both identify recent migrants, but while the GENODIVE analysis has the advantage of providing a formal likelihood ratio comparison, this test effectively assumes that identified individuals are 1^st^ generation migrants, a constraint that can lead to reduced sensitivity and mis-identification of the source population of 2^nd^ or 3^rd^ generation migrants.

In order to assess if the populations of *Arapaima* have experienced reductions in effective population size we used two moment-based methods implemented in the programs BOTTLENECK [45] and *M*Value [46], respectively. The program BOTTLENECK identifies populations that have experienced a reduction in effective population size by the presence of heterozygosity excess due to the loss of rare alleles, assuming an approximate infinite alleles model, wherein each mutation creates a new allele. The M-ratio implemented by *M*value, which considers the range of repeat numbers of microsatellite alleles relative to richness, is considered more sensitive to recent reductions in effective population size, but requires assuming that surveyed microsatellites evolve by quasi-stepwise mutation [46].

The two moment-based methods efficiently detect recent bottlenecks—population reductions with the last few generations; however, populations and species are also subject to historical demographic growth and/or reductions. Therefore we analyzed the data using the coalescent sampler implemented in the program MSVar v1.3 [47, 48]. We ran 10 independent parallel chains sampling every 1,000th proposal, collecting 20,000 proposals in the MCMC chain in each parallel run. Priors for current and historical population size means and variances were set equal, with variances encompassing three orders of magnitude. Prior for mean time of population size change was set at 1,000 generations ago with variance encompassing time range from 1,000,000 to 0 generations. The runs were evaluated for convergence and were pooled to provide an estimate of current and historical effective population size. Convergence was assessed using the Gelman–Rubin criterion [49] and the test of alternative hypotheses (population decline vs. stable population size) was carried out as suggested by Beaumont [47] using Bayes factors. Calculations and plots were performed in the R statistical programming language [40] using the packages CODA [50] and ggplot2 [51].

In addition to these tests, we also estimated the effective population size (*Ne*) for each population using the LDNe method [52] implemented in the program NeEstimator v2.0 [53], assuming a random mating model and allele frequencies cutoff of 0.02. This analysis, which estimates the number of individuals contributing to the sample based on allelic linkage, assumes that the sample is representative of the age structure of the population, and, when it is not, the Ne estimates are equivalent to the number of breeders that contributed offspring to the generations included the sample [54].

## Results

We surveyed genotypes of 11 microsatellite loci in a total of 511 individuals from the mainstem and major tributaries of the Amazon River, the Araguaia-Tocantins River, and Região dos Lagos (Fig 1). MICRO-CHECKER suggested there was no evidence of null alleles in the data. After Bonferroni correction, linkage disequilibrium was observed in 1 to 4% of pairwise comparisons for most loci; however, loci CTm3 and CTm4 had more than 30% probability of being linked. Locus CTm3 was also in H-W disequilibrium in 4 of 22 populations, and so this locus was removed from population structure analyses. Genetic statistics per locus and per sampling locality are shown in S1 Table. Overall, various diversity parameters presented low values for the eastern end of the Amazon Basin (Mexiana and Região dos Lagos), as well as in the middle and upper Purus River (Lábrea, Manuel Urbano), and upper Araguaia River (Ilha do Bananal, APA Meandros do Araguaia). Statistics of genetic diversity by sampling area are listed in Table 2, which shows that the average gene diversity over loci ranged from 0.128 ± 0.111 (APA Meandros do Araguaia) to 0.649 ± 0.342 (Mamirauá). The average number of alleles varied from 6.35 in Mamirauá to 2.17 in Ilha do Bananal (S1 Fig). The inbreeding coefficient, *F*_*IS*_, ranged from low in Nhamundá (0.00) to high in Ilha do Bananal (0.54) (Table 2); however, only 4 out of the 21 localities were significant (Santa Cruz, Manuel Urbano, and Manacapuru). Proportions of private alleles ranged from 0.02 in Região dos Lagos to 0.67 in Resex Unini, and eight localities presented frequencies of <0.10 of private alleles. Expected heterozygosity (*H*_*E*_) varied from 0.26 in the specimens collected in the Araguaia-Tocantins drainage (APA Meandros do Araguaia) to 0.66 in the specimens collected near the main channel, at Mamirauá (S2 Fig). Considering only mainstem locations, *H*_*E*_ ranged from 0.50 in the specimens collected in Mexiana to 0.66 in the specimens collected in Mamirauá. Conversely, the specimens collected in the tributaries of the Amazon basin showed *H*_*E*_ of 0.26 in the APA Meandros do Araguaia in the upper Araguaia River, and 0.62 in Tapauá, in the middle Purus River. Five loci were monomorphic for specimens collected at Ilha do Bananal (Araguaia River), two for specimens from the upper Purus River (Manuel Urbano) and one locus in the middle Purus River (Tapauá), Região dos Lagos (Amapá) and APA Meandros do Araguaia.

**Table 2:** Genetic characterization of *Arapaima gigas* sampled from 22 localities in Amazônia.

Localities	N	Average gene diversity over loci	N_A_	A_R_	P_A_	Average HWE H_O_−H_E_	*F*_*IS*_	Monomorphic loci
1. Santa Cruz	16	0.561 ± 0.304	5.45	3.78	0.27	0.449–0.561	0.201*	
2. Puerto Nariño	22	0.546 ± 0.295	5.27	3.63	0.18	0.503–0.546	0.079	
3. Carauari	18	0.514 ± 0.296	4.45	3.26	0.13	0.583–0.590	-0.021	
4. Eirunepé	13	0.594 ± 0.329	4.17	3.18	0.16	0.482–0.544	0.108	
5. Mamirauá	32	0.649 ± 0.342	6.35	3.95	0.20	0.695–0.660	-0.065	
6. Coari	9	0.602 ± 0.332	3.54	3.13	0.01	0.606–0.602	-0.007	
7. RDS Piagaçu-Purus	20	0.540 ± 0.296	4.54	3.23	0.05	0.586–0.569	-0.054	
8. Tapauá	20	0.547 ± 0.295	4.90	3.43	0.14	0.588–0.622	0.039	CAm20
9. Lábrea	15	0.343 ± 0.207	3.81	2.66	0.28	0.423–0.405	-0.075	
10. Manuel Urbano	18	0.421 ± 0.245	2.45	2.16	0.06	0.362–0.435	0.170*	CAm15, CAm20
11. Manacapuru	30	0.606 ± 0.321	6.27	3.57	0.15	0.554–0.620	0.098*	
12. Resex Unini	21	0.555 ± 0.310	5.27	3.60	0.67	0.558–0.617	-0.013	
13. Careiro da Várzea	21	0.559 ± 0.303	4.90	3.36	0.13	0.548–0.567	0.022	
14. Borba	30	0.540 ± 0.295	4.45	2.90	0.07	0.530–0.563	0.033	
15. Nhamundá	7	0.619 ± 0.350	3.81	3.44	0.24	0.606–0.606	-0.005	
16. Santarém	31	0.625 ± 0.330	5.54	4.57	0.10	0.608–0.635	0.036	
17. Jacareacanga	15	0.532 ± 0.302	3.81	2.93	0.12	0.632–0.556	-0.181	
18. Região dos Lagos	30	0.362 ± 0.204	2.81	2.32	0.02	0.431–0.407	-0.071	CTm5
19. Mexiana	17	0.489 ± 0.275	3.18	2.73	0.09	0.438–0.501	0.117	
20. Tucuruí	31	0.514 ± 0.277	3.54	2.65	0.02	0.478–0.515	0.072	
21. Ilha do Bananal	15	0.145 ± 0.107	2.17	1.57	0.04	0.647–0.459	-0.546	CAm16, CAm20, CTm3, CTm4, CTm8
22. APA Meandros do Araguaia	80	0.128 ± 0.111	3.10	1.79	0.10	0.258–0.262	-0.062	CAm15

Note: N = number of individuals analyzed; N_A_ = average number of alleles per locus; A_R_ = Allelic richness; P_A_ = Private Allelic richness; H_O_ = Observed heterozygosity; H_E_ = Expected heterozygosity; *F*_*IS*_ = Fisher’s individual fixation index (“inbreeding coefficient”)

* Indicates significant *P* value after Bonferroni correction.

### Distribution of genetic variability and population differentiation

**Fig 2:**
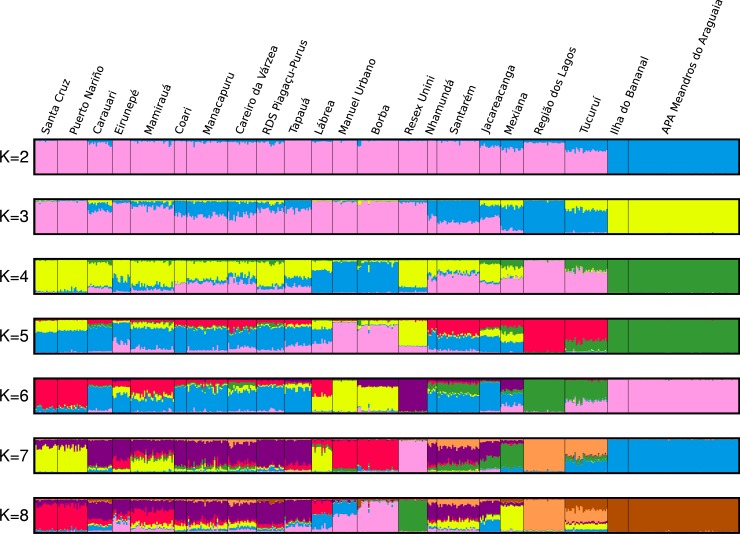
Graph of population structure of *Arapaima gigas* estimated in the program STRUCTURE. Each individual is represented by a vertical line.

**Fig 3:**
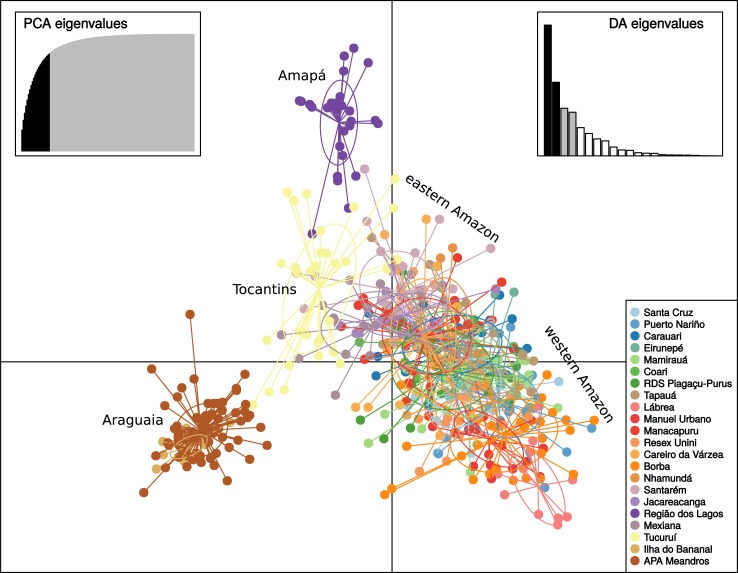
Results of DAPC analysis showing the scatterplot of the first two principal components based on 11 microsatellite loci of 511 individuals of *Arapaima gigas* from 22 sampling locations. The results are plotted showing an ellipse that shows one standard deviation of the variation of each population relative to its centroid. The eastern Amazon and western Amazon labels designate concentration of localities from these geographic regions.

Evaluation of clustering of individuals with STRUCTURE based on variance in likelihood among runs and across numbers of clusters (delta K) showed that two clusters (K = 2) was optimal, with an additional peaks at K = 3 and K = 6 (Fig 2 and S3 Fig). These clusters corresponded to geography. At K = 2, the clusters indicate the distinctness of fishes from the Araguaia-Tocantins system and its area of influence—and principally those of the upper Araguaia River (APA Meandros do Araguaia, Ilha do Bananal), and the rest of the Amazon basin. At K = 3, Amazonian populations show an east-west structuring gradient. At K = 6, it is also apparent that populations geographically distant from the mainstem of the Amazon River, or not directly connected to it, also show certain degree of reproductive divergence (Fig 2). At K = 6, STRUCTURE results emphasized the distinctness of location in the Purus drainage (Manuel Urbano, Lábrea), lower Madeira (Borba), the Negro (RESEX Unini), upper Tapajós (Jacareacanga) and Amapá (Região dos Lagos). Interestingly, SAMOVA with K = 2 or K = 3, emphasized the same groupings as STRUCTURE (Araguaia, Amapá), which explained 17% of the genetic variance (*F*_*CT*_ = 0.16748) by separating the Araguaia, and 18% (*F*_*CT*_ = 0.17894) by separating both (with p<0.004). However, at K = 4 and K = 5, SAMOVA grouped the Amazon delta (Mexiana) and Negro (Unini) separately, which only provided marginal increases in the genetic variance explained (*F*_*CT*_ = 0.18538 and 0.19299, respectively). Finally, the DAPC showed contiguous overlap among most localities with the exception of the Araguaia and Amapá, and with the Tucuruí locality intermediate between these three groups (Fig 3). The congruency of these analyses with groupings at K = 2 and 3 and incongruence at larger K values indicate the robustness of this population structure.

### Correlation between genetic divergence and geographic proximity

**Fig 4:**
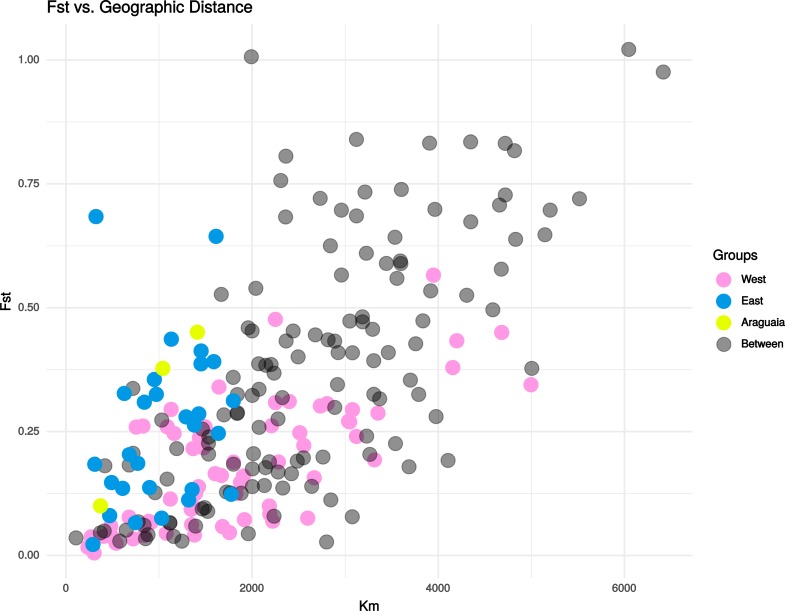
Graph of spatial autocorrelation analysis using linear pair-wise *F*_*ST*_ data and the distance between locations in kilometers (following the course of the rivers), all the distance categories showed significant correlations. The groups West, East and Araguaia refer to pair-wise comparisons between localities within each group, while Between are comparisons between localities of these groups. East and West localities are those that are east and west of the Madeira/Negro divide.

**Fig 5:**
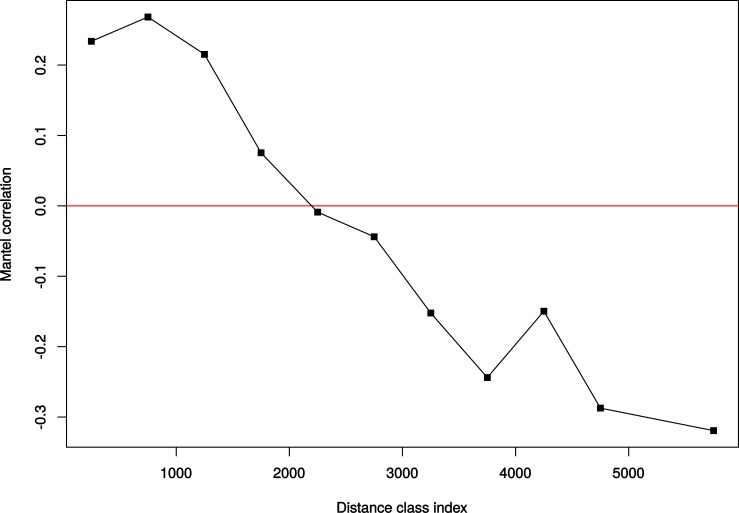
Graph of Mantel correlograms among localities.

Despite forming an evolutionarily coherent population, the genetic structure among localities in the larger, Amazonas basin group was not insignificant, and *F*_*ST*_ values ranged from 0.019 and non-significant (Alto Jurua x Nhamundá) to 0.475 and highly significant (*P*<0.0001; Lábrea x Região dos Lagos in Amapá) (S2 Table and S4 Fig). A Mantel test indicated that this pattern of genetic divergence was significantly predicted by distance (r = 0.618174, *P* = 0.0003), suggesting that isolation-by-distance processes structure genetic diversity at the largest scale in the Amazonas group. A Mantel test was similarly positive including all samples (r = 0.673128, *P* = 0.0001) (Fig 4). Mantel correlograms showed positive spatial autocorrelation in genetic distance among localities up to 1,500 km, while localities separated by more than 2,000 km showed negative or non-significant spatial autocorrelation (Fig 5), indicating that neutral evolutionary processes across populations are dominated by gene flow up to 1,500 km, after which genetic drift plays a larger role between most populations. Importantly, these patterns were true considering all samples or without the Araguaia or Amapá samples.

Analysis of gene flow implemented in the program MIGRATE 3.6.11 [43] revealed a mixed pattern of gene flow among populations. The result indicated bidirectional gene flow between all localities (S2 Table), with all cases presenting more than 1 individual per generation. Although it is clear the reduction in *Nm* values when the populations of the Araguaia River are included.

Likelihood tests in GENODIVE (Table 3) identified six migrants that were also corroborated by STRUCTURE: three of these with all migration priors, two with the two larger priors, and a single migrant only with the highest prior. Four additional individuals were identified by STRUCTURE with all priors as being 1^st^ or later generation migrants, and a single individual was identified by GENODIVE as a migrant but was not corroborated in any STRUCTURE run (not shown). Although several of these migrants were from adjacent localities (e.g. Manacapuru and Nhamundá), several also suggested relatively distant dispersals, albeit potentially over several generations.

**Table 3:** Results of Likelihood test implemented in GENODIVE.

Migrant	Sampled at	Assigned to*	Generation
1	Jacareacanga	Santarém	1st
2	Manacapuru	Nhamundá	1st
3	Careiro da Várzea	Mamirauá	1st
4	Tucuruí	Borba	3rd
5	Borba	Tapauá	1st
6	Tapauá	Eirunepé	1st
7	Lábrea	RDS Piagaçu-Purus	2nd
8	Santa Cruz	Lábrea	3rd
9	Borba	Santarém	2nd
10	Manacapuru	Santarém	3rd

*From STRUCTURE with migration prior 5x10^-2^.

### Evidence of population size changes

Results for analyses of a recent reduction in effective population size (bottleneck effect) are presented in Table 4. BOTTLENECK analyses detected significant deviations in observed heterozygosity in 12 groups of individuals. Reduction in number of alleles implemented in the program *M*Value also indicated that 12 *Arapaima* localities experienced a significant reduction in size (*M*<0.68), according to Garza & Williamson [46], eight of them from tributaries. Areas which showed significant reduction were not necessarily the same in both analysis. Additionally, estimates of effective number of breeders were relatively low for many sites (Table 4).

**Table 4:** Bottleneck metrics for *Arapaima gigas* by locality.

Map location	N	Localities	IAM	TPM	SMM	M value (*P*)	Ne (95% CI)
1	16	Santa Cruz	0.89844	0.41309	**0.02100**	0.776 (0.0494)	95 (23 –Inf)
2	22	Puerto Nariño	0.27832	0.96582	0.24023	0.742 (0.0093)	14.1 (9.3–23)
3	18	Carauari	**0.00342**	0.08301	0.36523	**0.623 (< 0.0001)**	7.4 (3.3–13.6)
4	13	Eirunepé	0.32031	0.63770	0.14746	**0.646 (0.0006)**	13.3 (4.7–103.3)
5	32	Mamirauá	**0.00049**	0.05371	0.17480	0.760 (0.0154)	21.6 (14.5–34.6)
6	9	Coari	0.05371	0.36523	0.89844	**0.632 (0.0001)**	41.8 (6.7 –Inf)
7	20	RDS Piagaçu-Purus	0.17481	0.32031	0.70020	**0.658 (< 0.0001)**	5.9 (3.0–10.0)
8	20	Tapauá	**0.03223**	0.69531	**0.01856**	0.705 (0.0017)	31.6 (15.1–173.3)
9	15	Lábrea	0.27832	0.14746	**0.02100**	**0.587 (< 0.0001)**	1.1 (0.8–1.5)
10	18	Manuel Urbano	0.20313	1.00000	0.65234	**0.646 (< 0.0001)**	1.5 (1.0–2.4)
11	30	Manacapuru	0.41309	0.46484	**0.00684**	0.757 (0.0123)	18.8 (12.4–30.7)
12	21	Resex Unini	0.20606	0.76465	0.57715	**0.658 (0.0001)**	21.9 (11.3–67.9)
13	21	Careiro da Várzea	0.27832	0.89844	0.12305	**0.670 (0.0004)**	10.2 (6.0–17.7)
14	30	Borba	**0.00342**	0.96582	**0.02100**	0.694 (0.0005)	23.8 (14.4–47.2)
15	7	Nhamundá	0.96582	0.57715	0.41309	**0.651 (0.0005)**	Inf (20.6—Inf)
16	31	Santarém	**0.01611**	0.89844	0.36523	0.755 (0.0131)	48.8 (25.8–160.1)
17	15	Jacareacanga	**0.01221**	0.46484	0.10156	0.686 (0.0013)	2.0 (1.5–2.9)
18	30	Região dos Lagos	0.10547	0.55664	0.49219	0.694 (0.0003)	11.8 (6.0–24.3)
19	17	Mexiana	**0.02100**	0.27832	0.70020	**0.576 (< 0.0001)**	14.8 (6.1–68.7)
20	31	Tucuruí	**0.03483**	0.34907	0.57445	0.735 (0.0151)	5.6 (3.1–10.9)
21	15	Ilha do Bananal	**0.03125**	**0.03125**	**0.04688**	**0.662 (0.0064)**	Inf (2.0-Inf)
22	80	APA Meandros do Araguaia	0.12891	**0.00977**	**0.00977**	**0.601 (< 0.0001)**	0.8 (0.5–1.2)
Total	511						

Note: N = number of individuals analyzed; IAM = Infinite Alleles Model; TPM = Two Phase Model; SMM = Stepwise Mutation Model; M value = ratio of number of alleles and allelic spread, see [46]; Ne = Effective population size estimated from linkage disequilibrium. Values in bold are significant. Significance of M values is based on simulations; however, empirical studies suggest that populations that have suffered recent bottlenecks have < 0.68 [46].

**Fig 6:**
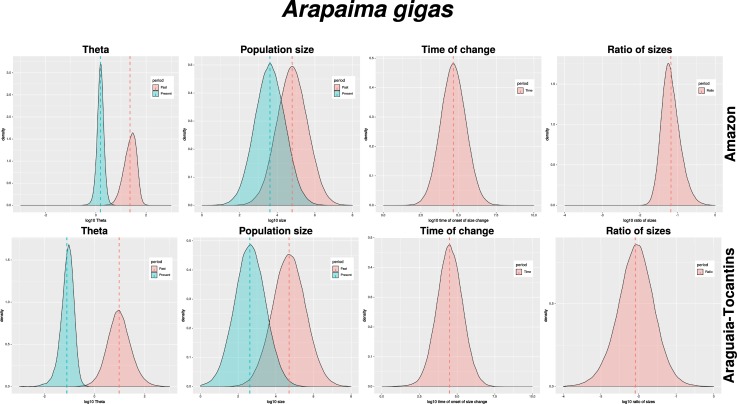
Coalescent population size change, Araguaia-Tocantins vs. Amazon. Note: all metrics are log10 scale. Theta–historical and current theta (4*Neμ*); Population size–historical and current effective population size (*Ne*); Time of change–onset of population size change from historical to current theta; Ratio of sizes–ratio of current to historical theta.

**Fig 7:**
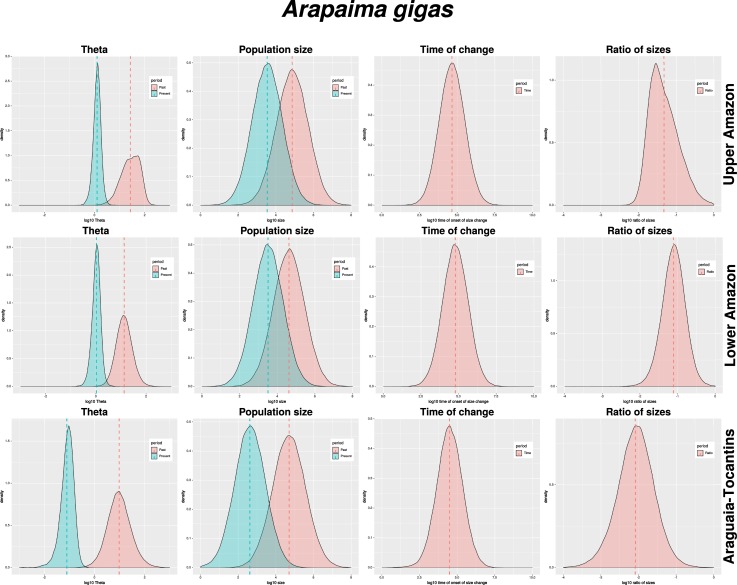
Coalescent population size change, Araguaia-Tocantins vs. lower Amazon vs. upper Amazon. Note: all metrics are log10 scale. Theta–historical and current theta (4*Neμ*); Population size–historical and current effective population size (*Ne*); Time of change–onset of population size change from historical to current theta; Ratio of sizes–ratio of current to historical theta.

In addition to recent population declines, coalescent analyses implemented in the program MSVar [55] indicate long-term decline as well (Figs 6 and 7 and Table 5). Analyses partitioned into two (Araguaia-Tocantins and Amazon) or three (Araguaia-Tocantins, lower Amazon and upper Amazon) groups show the same pattern. Historical population sizes of all groups were approximately equal (Ne 4.71, 95% HPD 3.26–6.15 vs. 4.62, 95% HPD 3.28–6.01 and 4.87, 95% HPD 3.51–6.27; Theta 0.98, 95% HPD 0.23–1.77 vs. 1.13, 95% HPD 0.59–1.65 and 1.43, 95% HPD 0.85–2.01) and began to decline at approximately the same time (4.49, 95% HPD 3.09–5.89 vs. 4.78, 95% HPD 3.39–6.15 and 4.65, 95% HPD 3.28–6.04). However, while populations declines centered on an order of magnitude in the Amazon basin (1.11, 95% HPD 1.62–0.60 and 1.33, 95% HPD 1.91–0.71), they were over two orders of magnitude in the Araguaia-Tocantins basin (2.09, 95% HPD 2.89–1.25).

**Table 5:** Population decline coalescent metrics for *Arapaima gigas* by region.

Region	Metric	log value (95% HPD)
Araguaia	Current *N*_*E*_	2.62 (1.27–3.99)
	Past *N*_*E*_	4.71 (3.26–6.15)
	Beginning of decline	4.49 (3.09–5.89)
	Current Theta	-1.10 (-1.50 –-0.65)
	Past Theta	0.98 (0.23–1.77)
	Magnitude of decline	-2.09 (-2.89 –-1.25)
Amazon	Current *N*_*E*_	3.61 (2.30–4.92)
	Past *N*_*E*_	4.79 (3.48–6.15)
	Beginning of decline	4.65 (3.29–6.03)
	Current Theta	0.18 (-0.04–0.40)
	Past Theta	1.36 (0.96–1.74)
	Magnitude of decline	-1.18 (-1.58 –-0.78)
lower Amazon	Current *N*_*E*_	3.51 (2.21–4.84)
	Past *N*_*E*_	4.62 (3.28–6.01)
	Beginning of decline	4.78 (3.39–6.15)
	Current Theta	0.02 (-0.26–0.30)
	Past Theta	1.13 (0.59–1.65)
	Magnitude of decline	-1.11 (-1.62 –-0.60)
upper Amazon	Current *N*_*E*_	3.54 (2.24–4.86)
	Past *N*_*E*_	4.87 (3.51–6.27)
	Beginning of decline	4.65 (3.28–6.04)
	Current Theta	0.10 (-0.15–0.35)
	Past Theta	1.43 (0.85–2.01)
	Magnitude of decline	-1.33 (-1.91 –-0.71)

Note: Beginning of decline is reported in years based on generation time of five years; Magnitude of decline is ratio of current and past Theta (and *N*_*E*_).

## Discussion

The arapaima is a charismatic fish of special cultural and socioeconomic significance to riverine communities of the Amazon, as well as occupying an apex ecological role in aquatic habitats [15, 16]. As obligate air breathing fish, an adaptation which allows them to exploit hypoxic floodplain environments, *Arapaima gigas* must regularly surface to renew the air in its highly vascularized swim bladder, but this dynamic also makes them especially vulnerable to human exploitation [56]. *Arapaima gigas* has been part of the diet of the riparian inhabitants of Amazonia since the early 18^th^ century [56, 57] and gradually gained significant commercial importance. However, catches began to decline at least as early as the 1960s, and by the 1980s *Arapaima gigas* was commercially extinct close to major urban centers [56, 58, 59]. In 1975, *Arapaima gigas* was listed in Appendix II of CITES (Convention on International Trade in Endangered Species) as a species not necessarily under threat of extinction, but for which commercial use must be controlled to avoid utilization incompatible with its survival; yet it is still considered data deficient.

### Population structure and isolation-by-distance in a complex river system

One of the most prominent uncertainties surrounding *Arapaima* is the number of species present in this genus and their distribution. Contrary to the assertions of Stewart [9] [10], we find no evidence for multiple species of *Arapaima* existing or co-existing in the Amazon basin and satellite river basins. Our sampling does not include, however, samples from the Rupununi, a floodplain in the headwaters of the Essequibo River, and an area of occurrence of *Arapaima arapaima* (Valenciennes, in Cuvier and Valenciennes, 1847). However, based on extensive ichthyofaunal sharing between the Essequibo and Branco (Amazon) basin—254 freshwater fish species representing ~73.8% of the total species sampled [60], and the mid-Pleistocene separation of these basins [61, 62], we view it unlikely that the population from the Rupununi is not *A*. *gigas*.

While we find no evidence for the existence of multiple species of arapaima in the Amazonian ecosystem, we observed population structuring. We found that the greatest structure in our genetic data reflected geographic disjunction of *A*. *gigas* in the upper Araguaia-Tocantins—a basin that become effectively isolated from the Amazon basin at the Plio-Pleistocene boundary [63], while the remaining fishes from the Amazonas basin reflected a single, albeit structured population along the east-west axis, with fishes of the Região dos Lagos in Amapá representing eastern-most extreme of this gradient. This was consistent between the STRUCTURE, SAMOVA, and DAPC analyses. While it may be tempting to hypothesize that the Araguaia-Tocantins populations reflect undescribed species, we note that the degree of genetic divergence of these populations (e.g. as measured by F_ST_) falls within the range of many widespread but cohesive species e.g. Hey & Pinho [64], and moreover, although contemporary gene flow between Araguaia-Tocantins and Amazon may be restricted, the admixture evident in localities in the eastern Amazon reflects historical gene flow between these areas (Fig 2). Thus, we continue to refer to all individuals as part of a single species (*Arapaima gigas* Schinz 1822), though we note that our sampling did not include the region from which *A*. *arapaima* is described (Guyana).

Within the Amazon—independent whether or not Amapá and/or Araguaia-Tocantins, the two regions not draining directly into the Amazon, were included—there was a strong and significant association between genetic divergence and geographical proximity, indicating that the attenuation of gene flow by distance (isolation-by-distance) is the major process structuring Amazon *A*. *gigas* populations on a large geographical scale. This agrees with the results of Hrbek et al. [24, 25] who surveyed additional loci but fewer and more distantly spaced localities. Intriguingly, positive spatial autocorrelation, reflecting the distance across which the homogenizing effects of gene flow are expected to dominate the diversifying effects of genetic drift, was significant up to 1500 km, a notable distance considering that adult *A*. *gigas* are known to be largely sedentary, only making small-scale annual migrations to and from the floodplain. We did, however, also discover a number of recent-generation migrants, some between relatively distant localities (Table 3), an inference supported by field and telemetry data [65]. If indeed adults are generally sedentary and show site fidelity, this may suggest an important role for juvenile dispersal as a means of conveying gene flow between sub-populations.

It would be convenient to assume that the weak population structure is the result of contemporary demographic and evolutionary processes as mediated by current landscape structure, but it is well known that the Amazon landscape itself has had a dynamic history. In the area occupied by the larger *A*. *gigas* population, the Amazonas River in its current west-to-east format is understood to have only formed around 10–11 million years ago (mya) with the breaching of the “Purus Arch”, a basement arch now largely buried and so named for its location along the Solimões near the Purus River [66, 67]. Prior to this breaching, the “Proto-Solimões” and western Amazon drained northward to the Caribbean and separately from more eastern Atlantic versants [67]. Indeed, previous studies have inferred an effect of the Purus arch not only on species distributions but on contemporary population structuring of widespread species as well. For example, Farias and Hrbek [68], in their analysis of the genus *Symphysodon*, inferred lineage distributions consistent with the Purus arch. Similarly, Willis et al. [69] discovered that genetic diversity in *Cichla monoculus* west of the Purus Arch was a subset of that found farther east, consistent with ancient east-to-west colonization. Importantly, *Arapaima* fossils similar to *A*. *gigas* have been discovered in the Miocene age La Venta formation of Colombia (~13 mya) [70], an area that would have been part of the northward-draining “Lago Pebas” system that shortly preceded the current west-to-east arrangement [66, 71]. So, *Arapaima* may have been present but separated on both sides of the Purus Arch, or colonized the eastern Amazon from the Lago Pebas system in the west. However, we saw no clear indications of diminished genetic diversity in eastern populations (apart from localized depletions discussed below), and plots of *F*_*ST*_ vs. geographic distance were fairly continuous among localities on either side of the Purus Arch (not shown). Thus, if the biogeographic history of *Arapaima* was significantly influenced by the Purus Arch, gene flow in the intervening period appears to have largely obscured these effects (see also Hrbek et al. [24]).

The degree of population genetic structure in *Arapaima gigas*, with significant genetic divergence among most localities (S2 Table), is notable for Amazonian fishes, whose continued study has revealed important variance in population structuring patterns. For example, examining the mitochondrial control region at locations on the Amazonas River mainstem, Santos et al. [72] and Farias et al. [73] found high genetic variability for tambaqui (*Colossoma macropomum*) and relatively low population structuring over vast distances. An analysis of nuclear microsatellites of *C*. *macropomum* populations from the Amazon mainstem and its main tributaties by Santos et al. [74] confirmed the mtDNA pattern, however, populations from tributaries and principally those close to headwaters also showed certain degree of differentiation. Similarly, Batista & Alves-Gomes [75], who examined the control region of the catfish *Brachyplatystoma rousseauxii*, also found high levels of genetic polymorphism and the absence of structuring. Similar patterns have been found with mtDNA or microsatellite markers for *Brachyplatystoma platynemum* [76], *Brycon amazonicus* [77], and *Prochilodus nigricans* [78]. Notably, these species share a migratory or semi-migratory and broadcast-spawning life history, with no parental care. In contrast, the patterns discovered for *A*. *gigas* are more similar to other species with sedentary adults, limited batch fecundity, and/or significant parental care, including the cichlids *Cichla* [79, 80] and *Symphysodon* [81] and the freshwater ray *Paratrygon aierba* [82]. Thus, it appears there is a general trend in which genetic variability and the degree of population structure is strongly determined by life history strategy, an observation that may assist in defining effective management strategies in the absence of more detailed information [78][83]. However, we recommend that additional studies to determine the contributions of variation in body size, habitat preferences, and biogeographic history would be prudent [84].

### Implications for *Arapaima* conservation in a threatened river system

The floodplain has been reported as the environment with the highest productivity in the Amazon drainage basin, and is the most common environment of the Solimões-Amazonas axis. The flood-ebb system of the flooded forest, or 'flood pulse', provides exceptional availability of diverse habitats and seasonal resource abundance [85]. However, the floodplain is also the most threatened habitat [86][87]. *Arapaima gigas* is a floodplain specialist, inhabiting lakes and lagoons that are connected by channels to the riverine network of the Amazonas basin. Although *A*. *gigas* is not a “migratory” species, these fishes do make small lateral movements through the network of lakes and channels of the Amazon floodplain, which provide abundant food for growing young [18]. These rich floodplains also provide important corridors for linear migration of individuals along and among river courses [18].

Although, as demonstrated here, while the major population structure of *Arapaima gigas* are the differences among the Araguaia and Amazon basins and the hydrologically isolated Região dos Lagos (Amapá), the Amazonas population is structured by isolation-by-distance at the largest scale—with the Região dos Lagos representing its eastern-most extreme, we discovered statistically significant genetic divergence (i.e. *F*_*ST*_) among most localities (S3 Table); in fact, few localities were not significantly divergent, although few *F*_*ST*_ values were greater than 0.2—at mutation-drift equilibrium equivalent to *Nm* = 1. These results indicate that at the smallest sampled scales, sub-populations of *A*. *gigas* are likely to be demographically somewhat independent, and cannot be assumed to compensate for exploitation or replenish one another over fishery-relevant timescales (e.g. Waples [88]). As such, *A*. *gigas* at these, and potentially smaller, spatial scales should be considered separate Management Units [89]. This would explain why population trends are different between the Araguaia-Tocantins and Amazon basins, and also some of the differences between the upper and lower Amazon basins.

In this context, the results indicating recent population declines (genetic bottlenecks) compounded onto long-term demographic declines potentially associated with the extent and distribution of Amazonian floodplains [90, 91] and low effective number of breeders is concerning (Table 4), since it appears that demographic recovery will largely depend on local recruitment rather than immigration. Indeed, the majority of localities exhibited effective population sizes well below the “50/500” threshold for limiting inbreeding depression (50) and loss of genetic diversity due to excessive genetic drift (500) [92], guidelines that have even been suggested as needing upward revision [93]. We note, however, that basing management goals from meta-analyses can be risky [94], and species with high parental investment and smaller lifetime fecundity (“K” or “equilibrium” strategists; [95, 96]) often have higher census to effective population size ratios. It is interesting to note recent indications that the socially monogamous *A*. *gigas* may regularly engage in polygamous reproduction [97], thereby reducing potential inbreeding and subsequent genetic erosion. Moreover, for species with sedentary adults and potentially significant small-scale spatial genetic (family) structure, it is unclear what an expected effective population size should be at any given spatial scale, and low numbers may be a natural aspect of species that otherwise effectively purge deleterious recessive alleles [98]. However, small populations with low growth rates may nonetheless also experience higher rates of population extirpation in the face of environmental variation [99], and these dynamics may be exacerbated by human exploitation [100]. Given this uncertainty, we suggest that studies examining the minimum viable population size for *A*. *gigas* are warranted.

To curb continuing population declines, in 2001 fishing of *Arapaima gigas* was banned by IBAMA (Brazilian Institute of Environment and Renewable Natural Resources), except in management areas such as the Mamirauá and Piagaçu-Purus Sustainable Development Reserves (RDS) where fishing is controlled, preventing local overexploitation [101, 102]. Additional successes in sustainable exploitation of *A*. *gigas* have been reported for community based management initiatives in which individual communities or families restrict access to spatially delimited populations of *A*. *gigas* e.g. [103]. Whether these successes can be replicated more widely and ensure long-term viability of *A*. *gigas* remains to be seen, especially with regard to evidence of low effective population sizes, genetic bottlenecks, and the potential for erosion of genetic diversity recovered here (Table 4). Whether or not these genetic patterns are the result of documented overexploitation, small populations depend on dispersal and immigration to maintain long-term genetic diversity e.g. [103]. Genetic diversity is the raw material upon which evolution acts, enabling populations to evolve in response to environmental changes, and without which a population may be more susceptible to extirpation or extinction [103, 104]. Here, our observation of recent migrants among populations is important, since it is these individuals that boost genetic variation in populations otherwise limited by local abundance. However, to effectively increase local diversity, these individuals must first successfully disperse. Despite localized sustainable initiatives and a ban on *A*. *gigas* fishing in Brazil, illegal fishing continues and creates risk even for dispersers among sustainably-managed areas. Moreover, the floodplain habitats of the Amazon basin through which these dispersers move have been reported as the most threatened in South America due to logging, forest clearing for cattle-ranching, construction of hydroelectric dams, and other disturbances [86]. The long-term viability of *A*. *gigas* fisheries will ultimately depend on addressing these significant regional challenges as well.

Some sub-populations of *Arapaima gigas* also face more localized hurdles to viability. We observed that statistics of genetic diversity were variable across localities, a result also observed by Hrbek et al. [24] with mtDNA. Intriguingly, although localities along the Amazon mainstem (e.g. Santarém, Carreiro da Varzea, Manacapuru, Coari) are those facing the greatest fishing pressure, they are also among the localities with the highest genetic diversity (Table 2), a feature we hypothesize to relate to their positions near the intersections of the river network. Despite their genetic diversity, continued illicit exploitation suggest that the longevity of these populations may depend on management regimes than ensure minimum viable populations. On the other hand, several locations in the upstream portions of tributaries (Eirunepé, Manuel Urbano, Meandros do Araguaia) exhibit lower genetic diversity, a worrisome trend considering that, even though these are the sub-populations that currently face lower fishing pressure, they are increasingly subjected to major habitat changes in Brazil's “arc of deforestation.” As such, these localities may be those for which inbreeding and loss of genetic diversity would be most problematic, and for which immigration may be the most beneficial. Thus, one size does not fit all, and management strategies for the management units will need to be tailored to local challenges.

### *Arapaima* from the unique extra-Amazon populations

Although the population in the Amazonas basin *sensu stricto* contains the majority of individuals and greatest fishing pressure, populations in the Aragauia-Tocantins and Amapá deserve special consideration. The divergence and genetic poverty of the *Arapaima gigas* in the Araguaia River (APA Meandros do Araguaia and Ilha do Bananal) observed here corroborate the findings of Vitorino et al. [105], who showed low values of genetic diversity and structuring between populations of four localities in Araguaia and Tocantins Rivers, and those of Hrbek et al. [25], who discovered a number of mtDNA haplotypes endemic to the Tocantins basin. The Araguaia-Tocantins River basin is connected to the Amazonas basin by uninterupted freshwater, albeit in the form of small meandering channels, and many researchers do not consider the Araguaia-Tocantins part of the Amazonas basin, since it drains primarily through the “Pará River” to the south of Marajó Island. However, as the presence of *A*. *gigas* in both basins implies, they share a close biogeographic history and exhibit similar icthyofaunas [106], along with several other adjacent Atlantic versants (e.g. Araguari, Oiapoque, Parnaiba). However, although numerous species are apparently distributed in both basins, several recent studies have shown that the Araguaia and/or Tocantins exhibit endemic lineages of fishes e.g. [69, 107, 108] and other aquatic organisms, including the Araguaian river dolphin *Inia araguaiaensis* [109]. The confirmation of an Araguaian population of *A*. *gigas* is in keeping with these trends.

The most likely feature promoting divergence of aquatic organisms in the upper Tocantins basin is, ironically, one that no longer exists, the Itaboca waterfalls that were submerged when the Tucuruí hydroelectric dam was constructed. As such, the Tucuruí population of *A*. *gigas*, which presented as a genetic intermediate between the Araguaia and eastern Amazon sub-population in STRUCTURE and DAPC analyses, presents something of a conundrum. The sampling locations in the upper Araguaia River (AEP Meandros do Araguaia and Ilha do Bananal), in addition to being about 1,340 and 890 km, respectively, from the reservoir, are also separated by numerous rapids which may limit the gene flow between these areas, implying that the pattern of admixture may be natural. However, the dam itself was also built downstream of the historical barrier (Itaboca), trapping some of the downstream fauna within the flooded region. Thus, the admixture of lower Amazon/lower Tocantins and upper Tocantins lineages may instead be an anthropogenic effect of reservoir construction. Indeed, several other studies have suggested that aquatic organisms of Tucuruí exhibit unique patterns of admixture or hybridization different from that of the lower Tocantins e.g. [69]. In the face of planned and ongoing construction of hydroelectric dams on numerous rivers in Brazil, this trend should serve as a cautionary tale.

The Araguaia-Tocantins basin is considered highly threatened not only by hydroelectric projects in the headwaters of tributary rivers, but it is also among the areas in Brazil with the highest rates of landscape modification for cattle ranching, road construction, and agriculture [22]. The unique population of *A*. *gigas* in this region, despite relatively low fishing pressure, should thus be considered highly threatened, especially considering these fishes exhibited the lowest observed levels of genetic diversity. Whether this reduced diversity results from natural (e.g. Pleistocene-age colonizations or bottlenecks) or anthropogenic effects, the ability of this unique population to remain viable in the face of ongoing habitat modifications should be closely monitored.

The population of *Arapaima gigas* from the Região dos Lagos in Amapá are significant as well. This region, which is technically connected to the Amazonas basin by freshwater from the Amazonas outflow, exhibits an icthyofauna with affinities both for that of the Amazon, as well as those of coastal Guyana drainages to the north [106]. The presence of *A*. *gigas* here is thus not a surprise, although, considering the strong and turbulent current that runs along the coast and probably limits dispersal, nor is their distinctness from the other populations. Fortunately, a significant portion of the Região dos Lagos is protected by a state park which limits exploitation, although it remains unknown if *A*. *gigas* from the Araguari River are part of this population as well. In either case, habitat degradation from cattle ranching and urban development continues to encroach upon the watersheds were this population is found, and being limited to such a relatively small area, this unique population could easily be placed at risk if current protections were to prove inadequate.

## Conclusions

The findings presented here should be seen as an important warning about the fragility of *Arapaima gigas* populations, given the evidence of reduced genetic capacity and the intersection of threats against them. These data should contribute toward the design of management and conservation programs for this species in the Amazon, Amapá, and Araguaia-Tocantins regions.

## Ethics statement

Permits for field collection and molecular analyses were given by IBAMA/CGEN 11325–1. This study involved no experimentation, manipulation or euthanasia of any animals on part of the researchers. We simply collected tissue samples of exemplars fished by local communities for food and as fishes were brought to market. In these instances our institution (UFAM) does not require IACUC approval. Finally, this research could not have been submitted to IACUC for analysis since this committee became operational only after the completion of this study.

## Supporting information

S1 Fig Graphic representation of the average number of alleles per population of *Arapaima gigas*. (EPS)

S2 Fig Graphic representation of expected heterozygosity (*H*_*E*_) per population of *Arapaima gigas*. (EPS)

S3 Fig Absolute values of the 2^nd^ order rate of change of mean likelihoods of each K. (EPS)

S4 Fig Graphic representation of pairwise *F*_*ST*_ values among groups of individuals of *Arapaima gigas*. (EPS)

S1 Table Characteristics of the 11 microsatellite loci analyzed for *Arapaima gigas* considering separately the groups of individuals grouped by collection site. N_A_ = Total number of alleles; A_R_ = Allelic richness; H_O_ = Observed Heterozygosity; H_E_ = Expeted Heterozygosity; mono = Monomorphic locus; * significant *P* value for deviation from HWE after Bonferroni correction (P = 0.00455).
(DOCX)

S2 Table Number of migrants per generation *Nm* = Mθ/2. Row localities are sending individuals, while column localities are receiving individuals. Locality codes are: 1- Santa Cruz, 2- Puerto Nariño, 3- Carauari, 4- Eirunepé, 5- Mamirauá, 6- Coari, 7- RDS Piagaçu-Purus, 8- Tapauá, 9- Lábrea, 10- Manuel Urbano, 11- Manacapuru, 12- Resex Unini, 13- Careiro da Várzea, 14- Borba, 15- Nhamundá, 16- Santarém, 17- Jacareacanga, 18- Região dos Lagos, 19- Mexiana, 20- Tucuruí, 21- Ilha do Bananal, 22- APA Meandros do Araguaia.
(XLSX)

S3 Table Matrix of pairwise *F*_*ST*_ values among localities sampled for *Arapaima gigas*. Note: significant differences at p < 0.05 and after Bonferroni correction are in bold.
(XLSX)
