# Supplementary material for: The largest fish in the world’s biggest river: Genetic connectivity and conservation of Arapaima gigas in the Amazon and Araguaia-Tocantins drainages
Source: PLoS One. 2019 Aug 16;14(8):e0220882. doi: 10.1371/journal.pone.0220882 (PMC6697350; doi:10.1371/journal.pone.0220882)
Supplement: S1 Table — NA = Total number of alleles; AR = Allelic richness; HO = Observed Heterozygosity; HE = Expeted Heterozygosity; mono = Monomorphic locus; * significant P value for deviation from HWE after Bonferroni correction (P = 0.00455). (DOCX) [file pone.0220882.s005.docx]

Supplemental Table 1 – Characteristics of the 11 microsatellite loci analyzed for *Arapaima gigas* considering separately the groups of individuals grouped by collection site. N_A_ = Total number of alleles; A_R_ = Allelic richness; H_O_ = Observed Heterozygosity; H_E_ = Expeted Heterozygosity; mono = Monomorphic locus; * significant *P* value for deviation from HWE after Bonferroni correction (P = 0.00455).

| **Localities** |  | **CTm3** | **CTm4** | **CTm5** | **CTm7** | **CTm8** | **CAm2** | **CAm13** | **CAm15** | **CAm16** | **CAm20** | **CAm26** |
| --- | --- | --- | --- | --- | --- | --- | --- | --- | --- | --- | --- | --- |
| **Santa Cruz** | N_A_ | 5 | 2 | 7 | 8 | 2 | 8 | 12 | 7 | 4 | 3 | 2 |
|  | A_R_ | 4.5 | 2 | 5.1 | 5.3 | 1.3 | 4.5 | 6.7 | 5.2 | 3.0 | 2.1 | 1.8 |
|  | H_O_ | 0.375 | 0.375 | 0.625 | 0.625 | 0.063 | 0.500 | 0.813 | 0.625 | 0.563 | 0.250 | 0.125 |
|  | H_E_ | 0.776 | 0.443 | 0.798 | 0.794 | 0.063 | 0.635 | 0.869 | 0.806 | 0.528 | 0.232 | 0.226 |
|  | *P* | **<0.001** | 0.592 | 0.385 | 0.181 | 1.000 | 0.186 | 0.367 | 0.129 | 0.558 | 1.000 | 0.191 |
| **Puerto Nariño** | N_A_ | 6 | 2 | 8 | 9 | 3 | 7 | 11 | 4 | 3 | 3 | 2 |
|  | A_R_ | 4.3 | 1.9 | 5.2 | 5.7 | 1.8 | 4.4 | 7.3 | 2.8 | 2.8 | 2.0 | 1.8 |
|  | H_O_ | 0.455 | 0.455 | 0.727 | 0.727 | 0.182 | 0.727 | 0.864 | 0.273 | 0.682 | 0.227 | 0.227 |
|  | H_E_ | 0.724 | 0.359 | 0.794 | 0.846 | 0.172 | 0.734 | 0.912 | 0.443 | 0.608 | 0.212 | 0.206 |
|  | *P* | **<0.001** | 0.537 | 0.123 | 0.096 | 1.000 | 0.226 | 0.027 | 0.018 | 0.811 | 1.000 | 1.000 |
| **Carauari** | N_A_ | 3 | 2 | 5 | 5 | 3 | 8 | 6 | 3 | 4 | 4 | 2 |
|  | A_R_ | 2.3 | 1.8 | 4.1 | 4.0 | 2.5 | 5.6 | 4.1 | 3.0 | 3.7 | 2.7 | 2.0 |
|  | H_O_ | 0.277 | 0.277 | 0.625 | 0.941 | 0.944 | 0.944 | 0.555 | 0.588 | 0.588 | 0.333 | 0.333 |
|  | H_E_ | 0.494 | 0.246 | 0.738 | 0.746 | 0.560 | 0.846 | 0.676 | 0.679 | 0.734 | 0.417 | 0.355 |
|  | *P* | 0.101 | 1.000 | 0.045 | 0.281 | 0.001 | 0.051 | 0.039 | 0.142 | 0.156 | 0.017 | 1.000 |
| **Eirunepé** | N_A_ | 2 | 2 | 7 | 7 | 4 | 5 | 5 | 4 | 5 | 2 | 3 |
|  | A_R_ | 2.0 | 1.9 | 4.8 | 4.6 | 3.1 | 4.3 | 3.0 | 2.8 | 3.8 | 2.0 | 2.7 |
|  | H_O_ | 0.231 | 0.166 | 0.692 | 0.538 | 0.538 | 0.615 | 0.363 | 0.462 | 0.769 | 0.462 | 0.462 |
|  | H_E_ | 0.471 | 0.289 | 0.763 | 0.757 | 0.532 | 0.794 | 0.337 | 0.443 | 0.686 | 0.369 | 0.542 |
|  | *P* | 0.091 | 0.255 | 0.187 | 0.001 | 1.000 | 0.207 | 1.000 | 0.069 | 0.807 | 1.000 | 0.574 |
| **Mamirauá** | N_A_ | 5 | 3 | 8 | 9 | 3 | 9 | 16 | 6 | 4 | 4 | 3 |
|  | A_R_ | 3.6 | 2.6 | 5.2 | 5.3 | 2.4 | 5.1 | 6.8 | 3.9 | 3.3 | 2.6 | 2.5 |
|  | H_O_ | 0.625 | 0.387 | 0.906 | 0.875 | 0.437 | 0.781 | 0.968 | 0.813 | 0.709 | 0.563 | 0.594 |
|  | H_E_ | 0.627 | 0.423 | 0.819 | 0.827 | 0.400 | 0.814 | 0.883 | 0.689 | 0.694 | 0.566 | 0.516 |
|  | *P* | 0.003 | 0.028 | 0.311 | 0.286 | 1.000 | 0.439 | 0.233 | 0.701 | 0.983 | 0.848 | 0.702 |
| **Coari** | N_A_ | 3 | 3 | 5 | 5 | 2 | 4 | 5 | 4 | 3 | 2 | 3 |
|  | A_R_ | 3.0 | 2.8 | 3.8 | 3.8 | 2.0 | 3.6 | 4.2 | 3.2 | 3.0 | 2.0 | 2.9 |
|  | H_O_ | 0.555 | 0.444 | 0.777 | 0.777 | 0.444 | 0.777 | 0.444c | 0.555 | 0.444 | 0.666 | 0.777 |
|  | H_E_ | 0.627 | 0.464 | 0.679 | 0.679 | 0.471 | 0.679 | 0.621 | 0.608 | 0.686 | 0.523 | 0.582 |
|  | *P* | 0.631 | 0.531 | 0.427 | 0.430 | 1.000 | 0.881 | 0.088 | 0.113 | 0.179 | 0.538 | 0.709 |
| **RDS Piagaçu-Purus** | N_A_ | 3 | 3 | 7 | 8 | 2 | 8 | 8 | 2 | 4 | 3 | 2 |
|  | A_R_ | 2.3 | 2.6 | 4.8 | 5.1 | 1.2 | 5.4 | 4.4 | 2.0 | 3.1 | 2.5 | 2.0 |
|  | H_O_ | 0.368 | 0.450 | 0.800 | 0.823 | 0.050 | 0.950 | 0.650 | 0.350 | 0.650 | 0.750 | 0.600 |
|  | H_E_ | 0.510 | 0.488 | 0.799 | 0.797 | 0.050 | 0.833 | 0.647 | 0.450 | 0.637 | 0.539 | 0.507 |
|  | *P* | 0.316 | 0.655 | 0.344 | 0.511 | 1.000 | 0.568 | 0.310 | 0.349 | 1.000 | 0.088 | 0.649 |
| **Tapauá** | N_A_ | 3 | 1 | 8 | 10 | 4 | 7 | 5 | 3 | 7 | 3 | 4 |
|  | A_R_ | 2.6 | 1.0 | 5.3 | 5.6 | 2.5 | 4.7 | 3.6 | 2.2 | 4.3 | 2.3 | 3.4 |
|  | H_O_ | 0.450 |  | 0.789 | 0.900 | 0.600 | 0.789 | 0.450 | 0.316 | 0.600 | 0.350 | 0.650 |
|  | H_E_ | 0.573 | mono | 0.815 | 0.825 | 0.479 | 0.757 | 0.529 | 0.351 | 0.738 | 0.522 | 0.629 |
|  | *P* | 0.108 |  | 0.175 | 0.117 | 0.214 | 0.262 | 0.134 | 0.605 | 0.048 | 0.154 | 0.822 |
| **Lábrea** | N_A_ | 3 | 2 | 6 | 6 | 2 | 7 | 6 | 3 | 3 | 2 | 2 |
|  | A_R_ | 1.7 | 1.4 | 3.7 | 4.2 | 1.8 | 3.9 | 3.5 | 2.4 | 2.9 | 2.0 | 1.8 |
|  | H_O_ | 0.133 | 0.066 | 0.333 | 0.428 | 0.200 | 0.400 | 0.333 | 0.785 | 1.000 | 0.769 | 0.200 |
|  | H_E_ | 0.131 | 0.066 | 0.542 | 0.645 | 0.186 | 0.510 | 0.459 | 0.547 | 0.659 | 0.517 | 0.186 |
|  | *P* | 1.000 | 1.000 | 0.009 | **<0.001** | 1.000 | 0.135 | 0.074 | 0.100 | 0.014 | 0.112 | 1.000 |
| **Manuel Urbano** | N_A_ | 2 | 1 | 5 | 3 | 1 | 3 | 3 | 4 | 3 | 2 | 2 |
|  | A_R_ | 1.8 | 1.0 | 3.1 | 2.6 | 1.0 | 2.3 | 2.3 | 3.5 | 2.8 | 1.3 | 2.0 |
|  | H_O_ | 0.277 |  | 0.555 | 0.388 |  | 0.222 | 0.529 | 0.611 | 0.277 | 0.058 | 0.333 |
|  | H_E_ | 0.246 | mono | 0.488 | 0.414 | mono | 0.427 | 0.490 | 0.716 | 0.595 | 0.058 | 0.488 |
|  | *P* | 1.000 |  | 0.210 | 0.137 |  | 0.031 | 0.515 | 0.005 | 0.001 | 1.000 | 0.315 |
| **Manacapuru** | N_A_ | 4 | 4 | 8 | 7 | 3 | 9 | 14 | 8 | 5 | 3 | 4 |
|  | A_R_ | 2.9 | 2.4 | 4.4 | 4.0 | 2.3 | 5.0 | 5.8 | 3.8 | 3.3 | 2.6 | 2.8 |
|  | H_O_ | 0.448 | 0.333 | 0.700 | 0.689 | 0.433 | 0.666 | 0.689 | 0.600 | 0.533 | 0.466 | 0.533 |
|  | H_E_ | 0.605 | 0.322 | 0.723 | 0.694 | 0.459 | 0.802 | 0.764 | 0.658 | 0.642 | 0.567 | 0.577 |
|  | *P* | 0.074 | 0.192 | 0.032 | 0.072 | 0.840 | 0.163 | 0.159 | 0.246 | 0.083 | 0.059 | 0.789 |
| **Resex Unini** | N_A_ | 3 | 5 | 7 | 12 | 7 | 6 | 7 | 3 | 2 | 4 | 2 |
|  | A_R_ | 2.5 | 4.2 | 4.0 | 5.5 | 5.6 | 4.2 | 4.3 | 2.6 | 1.7 | 3.0 | 2.0 |
|  | H_O_ | 0.524 | 0.550 | 0.381 | 0.947 | 0.923 | 0.857 | 0.800 | 0.381 | 0.190 | 0.368 | 0.550 |
|  | H_E_ | 0.516 | 0.779 | 0.595 | 0.791 | 0.846 | 0.741 | 0.744 | 0.577 | 0.177 | 0.519 | 0.501 |
|  | *P* | 1.000 | 0.002 | **<0.001** | **<0.001** | **<0.001** | 0.918 | 0.146 | 0.168 | 1.000 | 0.003 | 1.000 |
| **Careiro da Várzea** | N_A_ | 3 | 4 | 8 | 5 | 3 | 10 | 8 | 3 | 4 | 3 | 3 |
|  | A_R_ | 2.9 | 3.0 | 5.4 | 3.2 | 1.9 | 5.7 | 4.4 | 2.4 | 3.2 | 2.8 | 2.2 |
|  | H_O_ | 0.571 | 0.333 | 0.905 | 0.789 | 0.190 | 0.762 | 0.666 | 0.350 | 0.619 | 0.428 | 0.428 |
|  | H_E_ | 0.641 | 0.568 | 0.835 | 0.603 | 0.180 | 0.835 | 0.648 | 0.376 | 0.631 | 0.563 | 0.354 |
|  | *P* | 0.462 | 0.017 | 0.242 | 0.021 | 1.000 | 0.162 | 0.401 | 0.686 | 0.167 | 0.020 | 0.637 |
| **Borba** | N_A_ | 5 | 3 | 5 | 6 | 5 | 4 | 7 | 3 | 5 | 3 | 3 |
|  | A_R_ | 2.9 | 2.2 | 3.1 | 3.4 | 3.3 | 3.1 | 3.6 | 2.3 | 3.6 | 2.2 | 2.2 |
|  | H_O_ | 0.392 | 0.517 | 0.400 | 0.428 | 0.655 | 0.466 | 0.500 | 0.793 | 0.800 | 0.310 | 0.566 |
|  | H_E_ | 0.588 | 0.518 | 0.574 | 0.596 | 0.603 | 0.520 | 0.650 | 0.536 | 0.671 | 0.482 | 0.450 |
|  | *P* | 0.032 | 0.683 | 0.037 | 0.047 | 0.813 | 0.005 | 0.006 | **<0.001** | 0.173 | 0.042 | 0.343 |
| **Nhamundá** | N_A_ | 2 | 3 | 6 | 6 | 3 | 6 | 4 | 3 | 3 | 3 | 3 |
|  | A_R_ | 2.0 | 2.8 | 5.1 | 5.1 | 2.8 | 5.1 | 3.5 | 3.0 | 2.8 | 2.8 | 2.8 |
|  | H_O_ | 0.666 | 0.571 | 0.714 | 0.714 | 0.571 | 0.571 | 0.571 | 0.714 | 0.571 | 0.571 | 0.428 |
|  | H_E_ | 0.485 | 0.538 | 0.736 | 0.736 | 0.582 | 0.681 | 0.495 | 0.670 | 0.604 | 0.604 | 0.538 |
|  | *P* | 1.000 | 0.328 | 1.000 | 1.000 | 1.000 | 0.288 | 1.000 | 0.775 | 1.000 | 1.000 | 1.000 |
| **Santarém** | N_A_ | 3 | 4 | 7 | 7 | 3 | 9 | 9 | 5 | 8 | 3 | 3 |
|  | A_R_ | 2.9 | 2.9 | 3.6 | 3.6 | 2.7 | 6.0 | 5.0 | 3.1 | 4.3 | 2.8 | 2.4 |
|  | H_O_ | 0.709 | 0.645 | 0.500 | 0.548 | 0.581 | 0.733 | 0.548 | 0.677 | 0.516 | 0.709 | 0.516 |
|  | H_E_ | 0.626 | 0.613 | 0.634 | 0.634 | 0.459 | 0.850 | 0.778 | 0.607 | 0.636 | 0.603 | 0.543 |
|  | *P* | 0.523 | 0.939 | 0.225 | 0.294 | 0.279 | 0.041 | **<0.001** | 0.709 | 0.109 | 0.144 | 0.690 |
| **Jacareacanga** | N_A_ | 4 | 3 | 4 | 3 | 3 | 5 | 3 | 6 | 5 | 4 | 3 |
|  | A_R_ | 3.2 | 2.4 | 2.7 | 2.4 | 2.7 | 3.7 | 2.2 | 4.5 | 3.6 | 2.4 | 2.4 |
|  | H_O_ | 0.462 | 0.800 | 0.800 | 0.714 | 0.600 | 0.867 | 0.133 | 0.643 | 0.600 | 0.467 | 0.867 |
|  | H_E_ | 0.603 | 0.549 | 0.545 | 0.500 | 0.476 | 0.713 | 0.297 | 0.754 | 0.646 | 0.480 | 0.549 |
|  | *P* | 0.201 | 0.064 | 0.111 | 0.222 | 0.575 | 0.002 | 0.020 | 0.596 | 0.351 | 0.466 | 0.016 |
| **Região dos Lagos** | N_A_ | 4 | 2 | 2 | 3 | 3 | 6 | 3 | 4 | 1 | 2 | 2 |
|  | A_R_ | 3.0 | 1.7 | 1.9 | 2.2 | 2.0 | 4.7 | 2.2 | 3.2 | 1.0 | 1.5 | 2.0 |
|  | H_O_ | 0.667 | 0.200 | 0.333 | 0.333 | 0.333 | 0.862 | 0.267 | 0.793 |  | 0.333 | 0.500 |
|  | H_E_ | 0.599 | 0.183 | 0.282 | 0.336 | 0.287 | 0.765 | 0.474 | 0.644 | mono | 0.097 | 0.413 |
|  | *P* | 0.133 | 1.000 | 0.563 | 0.014 | 1.000 | 0.628 | 0.016 | 0.312 |  | 0.051 | 0.376 |
| **Mexiana** | N_A_ | 3 | 2 | 4 | 4 | 2 | 4 | 4 | 4 | 4 | 2 | 2 |
|  | A_R_ | 2.5 | 1.4 | 3.5 | 3.5 | 1.7 | 3.8 | 3.3 | 3.4 | 2.7 | 2.0 | 2.0 |
|  | H_O_ | 0.647 | 0.118 | 0.647 | 0.588 | 0.176 | 0.687 | 0.471 | 0.588 | 0.313 | 0.176 | 0.412 |
|  | H_E_ | 0.554 | 0.114 | 0.677 | 0.683 | 0.166 | 0.732 | 0.683 | 0.583 | 0.381 | 0.487 | 0.451 |
|  | *P* | 0.215 | 1.000 | 0.582 | 0.248 | 1.000 | 0.188 | 0.098 | 0.326 | 0.085 | 0.012 | 1.000 |
| **Tucuruí** | N_A_ | 4 | 2 | 6 | 6 | 2 | 5 | 4 | 3 | 2 | 4 | 2 |
|  | A_R_ | 2.6 | 2.0 | 3.5 | 3.5 | 1.2 | 3.9 | 3.1 | 3.0 | 1.3 | 3.0 | 2.0 |
|  | H_O_ | 0.548 | 0.484 | 0.677 | 0.677 | 0.032 | 0.548 | 0.613 | 0.677 | 0.065 | 0.355 | 0.581 |
|  | H_E_ | 0.549 | 0.508 | 0.654 | 0.659 | 0.032 | 0.715 | 0.670 | 0.673 | 0.063 | 0.668 | 0.465 |
|  | *P* | 0.859 | 1.000 | 0.353 | 0.499 | 1.000 | 0.008 | 0.387 | 0.377 | 1.000 | 0.004 | 0.242 |
| **Ilha do Bananal** | N_A_ | 1 | 1 | 2 | 2 | 1 | 2 | 2 | 2 | 1 | 1 | 3 |
|  | A_R_ | 1.0 | 1.0 | 2.0 | 2.0 | 1.0 | 2.0 | 2.0 | 1.9 | 1.0 | 1.0 | 2.5 |
|  | H_O_ |  |  | 1.000 | 1.000 |  | 0.533 | 0.500 | 0.266 |  |  | 0.583 |
|  | H_E_ | mono | mono | 0.518 | 0.517 | mono | 0.405 | 0.516 | 0.239 | mono | mono | 0.562 |
|  | *P* |  |  | **<0.001** | **<0.001** |  | 0.507 | 1.000 | 1.000 |  |  | 1.000 |
| **APA Meandros do Araguaia** | N_A_ | 3 | 3 | 3 | 2 | 1 | 3 | 5 | 3 | 3 | 3 | 3 |
|  | A_R_ | 1.4 | 1.2 | 2.1 | 1.9 | 1.0 | 2.0 | 2.6 | 2.2 | 1.2 | 1.9 | 2.1 |
|  | H_O_ | 0.013 | 0.014 | 0.381 | 0.347 |  | 0.250 | 0.405 | 0.480 | 0.038 | 0.000 | 0.658 |
|  | H_E_ | 0.089 | 0.041 | 0.332 | 0.307 | mono | 0.228 | 0.436 | 0.434 | 0.038 | 0.202 | 0.508 |
|  | *P* | **<0.001** | 0.007 | 0.533 | 0.437 |  | 1.000 | 0.003 | 0.025 | 1.000 | **<0.001** | 0.013 |
